# Cardiac Magnetic Resonance, Electromechanical Activation, Kidney Function, and Natriuretic Peptides in Cardiac Resynchronization Therapy Upgrades

**DOI:** 10.3390/jcdd10100409

**Published:** 2023-09-22

**Authors:** Derek J. Bivona, Pim J. A. Oomen, Yu Wang, Frances L. Morales, Mohamad Abdi, Xu Gao, Rohit Malhotra, Andrew Darby, Nishaki Mehta, Oliver J. Monfredi, J. Michael Mangrum, Pamela K. Mason, Wayne C. Levy, Sula Mazimba, Amit R. Patel, Frederick H. Epstein, Kenneth C. Bilchick

**Affiliations:** 1 Department of Cardiovascular Medicine, University of Virginia Health System, Charlottesville, VA 22908, USA; djb6ab@uvahealth.org (D.J.B.); fmorales3@mgh.harvard.edu (F.L.M.); rm5s@uvahealth.org (R.M.); aed6d@uvahealth.org (A.D.); ojm9w@uvahealth.org (O.J.M.); jmm5v@uvahealth.org (J.M.M.); pkm5f@uvahealth.org (P.K.M.); sm8sd@uvahealth.org (S.M.); arp3d@uvahealth.org (A.R.P.); 2 Department of Biomedical Engineering, University of California Irvine, Irvine, CA 92617, USA; poomen@uci.edu; 3 Department of Biomedical Engineering, University of Virginia Health System, Charlottesville, VA 22908, USA; yw8za@virginia.edu (Y.W.); arya-abdi@siemens-healthineers.com (M.A.); fhe6b@virginia.edu (F.H.E.); 4 Department of Medicine, Northwestern University, Chicago, IL 60611, USA; xugao8@gmail.com; 5 Department of Medicine, William Beaumont Oakland University School of Medicine, Royal Oak, MI 48309, USA; nishaki.mehta@beaumont.org; 6 Department of Medicine, University of Washington, Seattle, WA 98195, USA; levywc@uw.edu; 7 Department of Radiology and Medical Imaging, University of Virginia Health System, Charlottesville, VA 22908, USA

**Keywords:** cardiac magnetic resonance, heart failure, cardiac resynchronization therapy, pacemakers, natriuretic peptides

## Abstract

As the mechanism for worse prognosis after cardiac resynchronization therapy (CRT) upgrades in heart failure patients with RVP dependence (RVP-HF) has clinical implications for patient selection and CRT implementation approaches, this study’s objective was to evaluate prognostic implications of cardiac magnetic resonance (CMR) findings and clinical factors in 102 HF patients (23.5% female, median age 66.5 years old, median follow-up 4.8 years) with and without RVP dependence undergoing upgrade and de novo CRT implants. Compared with other CRT groups, RVP-HF patients had decreased survival (*p* = 0.02), more anterior late-activated LV pacing sites (*p* = 0.002) by CMR, more atrial fibrillation (*p* = 0.0006), and higher creatinine (0.002). CMR activation timing at the LV pacing site predicted post-CRT LV functional improvement (*p* < 0.05), and mechanical activation onset < 34 ms by CMR at the LVP site was associated with decreased post-CRT survival in a model with higher pre-CRT creatinine and B-type natriuretic peptide (AUC 0.89; *p* < 0.0001); however, only the higher pre-CRT creatinine partially mediated (37%) the decreased survival in RVP-HF patients. In conclusion, RVP-HF had a distinct CMR phenotype, which has important implications for the selection of LV pacing sites in CRT upgrades, and only chronic kidney disease mediated the decreased survival after CRT in RVP-HF.

## 1. Introduction

Between 1993 and 2009, the number of patients implanted with permanent pacemakers increased by 55.6% in the United States, and 2.9 million patients overall received permanent pacemakers during this period [1]. Although conduction system pacing is increasing in popularity, RV pacing (RVP) is still more commonly implemented using active or passive fixation leads implanted in the ventricular septum, typically near the RV apex, which leads to the need for cardiac resynchronization therapy [2,3,4,5,6,7,8,9,10,11,12,13,14,15,16,17,18,19,20] in patients who develop heart failure [21]. Unfortunately, several studies have demonstrated worse survival after CRT upgrades for heart failure associated with RVP (RVP-HF) compared with survival in patients undergoing de novo CRT implants [22,23], although this finding has not been consistent across all studies [24,25]. Chronic kidney disease and atrial fibrillation in patients with CRT upgrades have been suggested as possible explanations for this difference in prognosis [22,23].

As most cardiac implantable electronic devices are now MR-conditional [26,27], and cardiac magnetic resonance (CMR) has been shown to provide important prognostic data in patients with heart failure [28,29], including those undergoing CRT [30,31], we designed a clinical study to evaluate differences in clinical and CMR parameters with respect to prognosis in patients undergoing CRT defibrillator upgrades for RVP-HF, CRT defibrillator upgrades for other indications, and de novo CRT defibrillator implants. The hypothesis was that selected CMR and clinical findings would be different in these patient groups and provide a mechanism for the decreased survival observed in multiple cohorts of patients undergoing CRT upgrade implants compared with those undergoing de novo CRT implants [22,23].

## 2. Materials and Methods

This study was approved by the Institutional Review Board for Human Subjects Research at the University of Virginia and included the enrollment of patients in different cohorts. Inclusion criteria for Group 1 required that patients were undergoing a de novo CRT defibrillator (CRT-D), and inclusion criteria for CRT upgrade patients required that they were undergoing an upgrade to a CRT defibrillator system with a coronary venous LV pacing lead for either RVP-HF (Group 2) or another accepted indication (Group 3). Other inclusion criteria for all patients were that they met published criteria for CRT [32] and were willing to have a CMR prior to the CRT procedure. The GFR had to be 45 cc/min/m^2^ before CRT to have a gadolinium-based contrast agent (GBCA) for research CMR at our institution, and the GBCA was withheld in the case of a lower GFR at the time of the scan. Exclusion criteria included contraindications for MRI, persistent atrial fibrillation without third-degree AV block, and a PVC burden greater than 15%. Patients were enrolled between 2011 and 2021.

Prior to CRT device implantation, clinical characteristics for all patients were collected through intake forms and cross-referenced with electronic health records. These included demographics, medications, comorbid conditions, the QRS duration from the 12-lead electrocardiogram, and relevant laboratory findings, as described in Table 1. Clinical parameters were also integrated as the Seattle Heart Failure Model score, as previously described [33].

Before the CRT procedure, patients underwent cardiac magnetic resonance (CMR) on a 1.5 T scanner. The MRI protocol included cine imaging, late gadolinium enhancement for myocardial scar detection, and strain imaging with displacement encoding with stimulated echoes (DENSE). Late gadolinium enhancement (LGE) was performed ten minutes after the injection of 0.1 mmol/kg of gadoterate meglumine (Dotarem), and a wideband pulse (bandwidth of 3.8 kHz) was added as needed [34] to minimize off-resonance artifacts in patients with cardiac implantable electronic devices at the time of the scan. Volumetric and scar analyses of CMR images were performed using suiteHEART Software v5.1.0 (Neosoft, Pewaukee, WI, USA).

Regional circumferential strain was acquired using cine DENSE [35] in short-axis planes at basal, mid-ventricular, and apical levels using a temporal resolution of 17 ms, pixel size of 2.8 × 2.8 mm^2^, and slice thickness of 8 mm. Displacement was encoded in two orthogonal directions, and a spiral k-space trajectory was used with 6 interleaves per image. Other parameters included: field of view = 350 × 350 mm^2^, displacement encoding frequency ke = 0.1 cycles/mm, flip angle = 15°, and echo time = 1.9 ms. DENSE images were analyzed using custom software. In a small number of patients in whom off-resonance artifacts were present on DENSE imaging, circumferential strain (E_cc_) was derived using a convolutional neural network (Strain-Net) designed by our group to predict regional strain from cine imaging and validated in a recently published study demonstrating more accurate regional strain assessments compared with commercially available feature tracking [36].

The CURE-SVD, a robust and validated mechanical dyssynchrony parameter (range: 0–1; 1 = greatest synchrony) was derived from E_cc_ as previously reported [37,38]. Regional mechanical activation was derived from CMR mechanical activation maps, which were created as previously reported for all patients from a regional E_cc_ matrix [30,37,38]. This matrix was derived from midwall E_cc_ based on cardiac segment and phase, and an active contour guided by the E_cc_ gradient was used to automatically detect the mechanical activation time field, which was defined as the time to the onset of circumferential shortening [38]. Intuitively, this is the time to the detection of a negative slope in a regional E_cc_ curve, as a negative slope of the E_cc_ curve indicates myocardial shortening. In order to calculate mechanical activation at the left ventricular pacing (LVP) site, the lead location was mapped to an LV segment (American Heart Association 17-segment model) based on procedural fluoroscopy using previously described methods [30,39,40,41].

Echocardiography was used to calculate the fractional change in left ventricular end-systolic volume index (LVESVI-FC) as: (LVESVI_Post-CRT_ − LVESVI_Pre-CRT_)/LVESVI_Pre-CRT_; negative values are favorable, and −0.15 is often used as a cutoff for response in the literature when there is a need to convert the LVESVI-FC to a binary response indicator [31]. Cardiopulmonary exercise testing measured peak VO_2_ before and after CRT, and measurements of BNP were used to consider the neurohormonal axis in CRT response.

Patients were followed for a median of 4.8 years for all-cause mortality up until 1 April 2023 through a review of the electronic health record. All patients enrolled were followed at the University of Virginia Health System, and their vital status was maintained in the electronic health record.

All statistical analyses were performed using R and Python. Statistical tests were performed to identify differences in baseline characteristics between the three upgrade/de novo CRT groups. Kruskal–Wallis tests were used to compare continuous variables, as not all data were normally distributed, and Dunn’s post hoc nonparametric test was used for pairwise comparisons. Chi-square tests and Fisher’s exact tests were used to compare differences in discrete variables. Reverse Kaplan–Meier curves stratified by cohort group were constructed, and the log-rank *p*-values were calculated. Median follow-up was determined based on the reverse Kaplan–Meier analysis. Logistic regression with least absolute shrinkage and selection operator for L1-regularization (LASSO logistic regression) was performed using the glmnet package to identify the predictors of survival. The tuning parameter λ was used based on expert recommendations [42] to eliminate all but four covariates. The model was then derived using logistic regression in a 70% training set from the cohort, and prediction accuracy was evaluated based on the model derived in the training set in a 30% test cohort. Survival at 4 years was chosen as the outcome for this logistic regression model based on the median follow-up time. Receiver operating characteristic (ROC) curves were plotted for performance in the model in the combined training and test sets with exact Pearson/Clopper 95% confidence intervals at three points of interest. A nomogram was also generated for the overall logistic regression model with four covariates for 4-year survival. These covariates were also evaluated in Cox proportional hazards models, for which hazard ratios, 95% confidence intervals, and *p*-values were reported.

Mediation analysis was performed to determine the extent to which the effect of an independent variable (the device group in this study) was mediated by predictors such as impaired renal function. The proposed mediator (creatinine) was regressed on the independent variable using linear regression, and survival/survival time was regressed on the mediator using a parametric survival model with a Gaussian distribution. The average causal mediation effect (ACME) was determined as the product of the regression coefficient for the effect of the independent variable on the mediator in the linear regression model and the regression coefficient for the effect of the mediator on survival time in 1000 bootstrapped samples. The method then facilitated the calculation of 95% confidence intervals for the mediator effect. This mediator effect was compared with the effect of the independent variable on survival/survival time.

## 3. Results

### 3.1. Baseline Characteristics and Response Measures of Entire Patient Cohort

The baseline characteristics for the 102 patients (median age 66.5 years old with interquartile range [IQR] 58.3 to 72.9; 23.5% female) are shown in Table 1.

**Figure 1:**
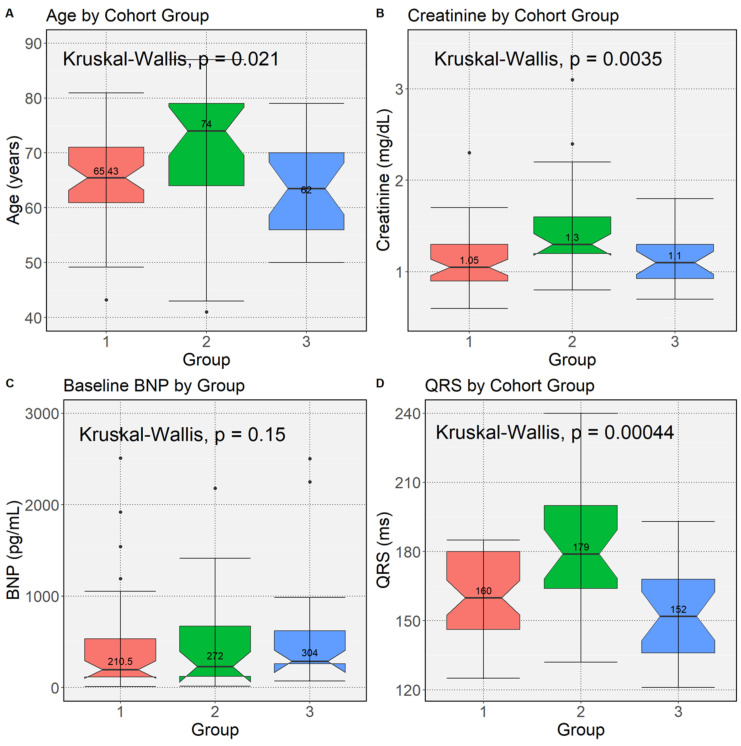
Box Plots for Clinical Characteristics by Upgrade Group. Box plots for differences in Groups 1–3 based on the Kruskal–Wallis test are shown for: (**A**) age (*p* = 0.021); (**B**) pre-CRT Creatinine (*p* = 0.0035); (**C**) pre-CRT BNP (*p* = 0.15); and (**D**) pre-CRT QRS Duration (*p* = 0.00044). BNP = B-type natriuretic peptide; CRT = cardiac resynchronization therapy.

During a median follow-up of 4.8 years (IQR 4.2 to 5.0 years), 26 (25.5%) patients died. The 4-year death rate was 18.7% (19/102). Group 2 patients were older (*p* = 0.03), had a higher frequency of atrial fibrillation (*p* = 0.0006), had greater pre-CRT QRS durations (*p* = 0.002), and had higher creatinine values (*p* = 0.002). BNP values were not significantly different across groups, although there was a trend for greater BNP in Group 2 vs. Group 1 (Figure 1).

### 3.2. Mechanical and Electrical Activation at the Left Ventricular Pacing Site by Upgrade/De Novo Group

**Figure 2:**
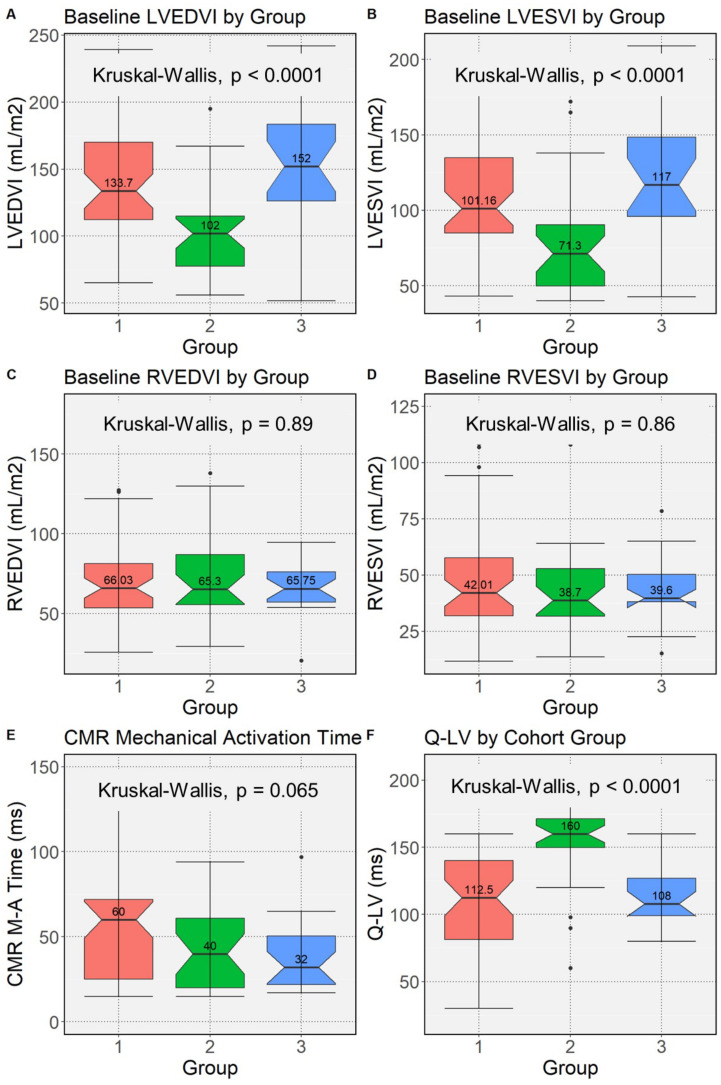
Box Plots for CMR and Lead Implant Findings by Upgrade Group. Box plots for differences in Groups 1–3 based on the Kruskal–Wallis test are shown for: (**A**) pre-CRT LVEDVI (*p* < 0.0001); (**B**) pre-CRT LVESVI (*p* < 0.0001); (**C**) pre-CRT RVEDVI (*p* = 0.89); (**D**) pre-CRT RVESVI (*p* = 0.86); (**E**) frequency of CMR mechanical activation time ≥34 ms at the LVP site (*p* = 0.065); and (**F**) Q-LV (*p* < 0.0001). CRT = cardiac resynchronization therapy. LVEDVI/LVESVI = left ventricular end diastolic/systolic volume index.

With respect to CMR structural findings, in comparison with Group 1, Group 2 patients had a significantly smaller baseline LVESVI (*p* = 0.0005), a smaller baseline LVEDVI (*p* = 0.0002), and greater LVEF (*p* = 0.06) (Figure 2).

**Figure 3:**
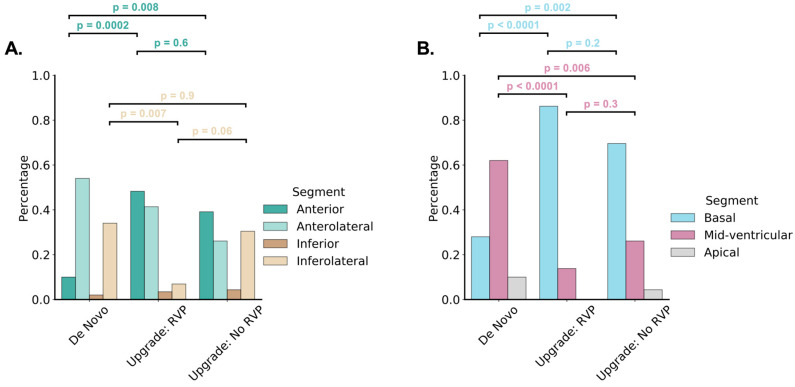
Distribution of Latest CMR LV Mechanical Activation by Group. The distribution of regions by group for late activation for the short-axis LV plane are shown with grouping for anterior, anterolateral, inferolateral, and inferior regions (**A**) and as basal, mid-ventricular, and apical regions (**B**).

The proportion with the favorable finding of mechanical activation greater than 34 ms at the LVP site was similar in all three groups (*p* = 0.31 vs. Group 1 and *p* = 0.80 vs. Group 3). (The cutoff of 34 ms was identified as the optimal multiple of 17 ms [DENSE temporal resolution] based on the Youden index to maximize sensitivity and specificity for 4-year survival.) As shown in Table 2 and Figure 3, Group 2 patients had a prominent anterior shift of late-activated sites compared with Group 1 (48% vs. 10% anterior, 42% vs. 52% anterolateral, and 10% vs. 38% inferolateral; *p* = 0.0002) with a similar distribution of LV free wall scar (30% vs. 17%, *p* = 0.2).

In separate linear regression models in the combined cohort of de novo CRT and CRT upgrade patients, a longer Q-LV time was associated with a greater improvement in the LVESVI after CRT (*p* = 0.020) after adjustment for CURE-SVD (*p* = 0.0085), which was also a significant predictor of this short-term outcome. LV free wall scar with LGE was associated with this response indicator alone (*p* = 0.057), but not after adjustment for either the Q-LV, the CURE-SVD, or both. For the outcome of improvement (lowering) in BNP post-CRT with adjustment for the pre-CRT value, both CURE-SVD (*p* = 0.037) and mechanical activation ≥ 34 ms at the LV lead implant site (*p* = 0.042) were significant predictors in a multivariable linear regression model. Both the Q-LV (*p* = 0.043) and CMR mechanical activation (*p* = 0.0039) were independently associated with survival time after CRT in a multivariable Cox proportional hazards regression model; however, only the mechanical activation predictor was associated with survival time after CRT after adjustment for pre-CRT BNP. Lead placement in anterior, anterolateral, posterolateral, or mid-apical-lateral AHA segments did not have an additional association in any of these models. No interactions with group assignments were noted in any of the models.

### 3.3. All-Cause Mortality by Group

**Figure 4:**
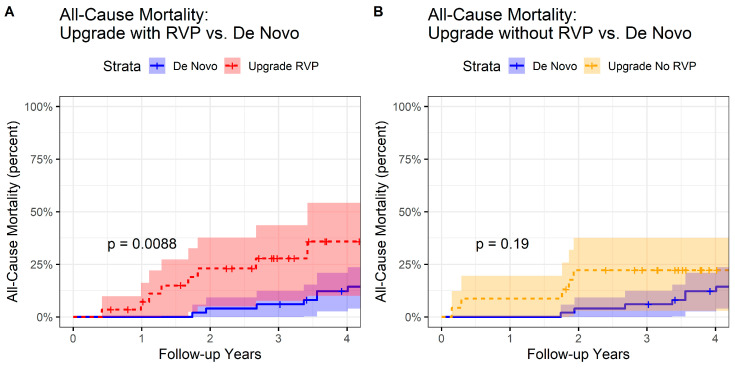
Reverse Kaplan–Meier Curves for CRT Upgrade and De Novo CRT Groups. Reverse Kaplan–Meier curves are shown for (**A**) CRT upgrade patients with RVP-HF (Group 2) versus de novo CRT patients (Group 1), and (**B**) CRT upgrade patients with other accepted indications (Group 3) versus de novo CRT Patients (Group 1). CRT = cardiac resynchronization therapy; RVP-HF = right ventricular pacing with heart failure.

In a Cox proportional hazards model, Group 2 patients had worse survival compared with Group 1 patients (HR 3.26 [95% CI 1.18–9.04], *p* = 0.023) (Figure 4A) but similar survival compared with Group 3 patients (Figure 4B). Comparing Group 1 and Group 2 patients, the six-month LVESVI-FC post-CRT (*p* = 0.006), six-month BNP post-CRT (*p* = 0.002), and six-month change in peak VO_2_ post-CRT (*p* = 0.02) were associated with long-term survival after adjustment for CRT group (*p* = 0.0002, *p* < 0.0001, and *p* = 0.002, respectively, in three separate models).

### 3.4. L1-Regularization for Selection of Covariates Associated with Survival

**Figure 5:**
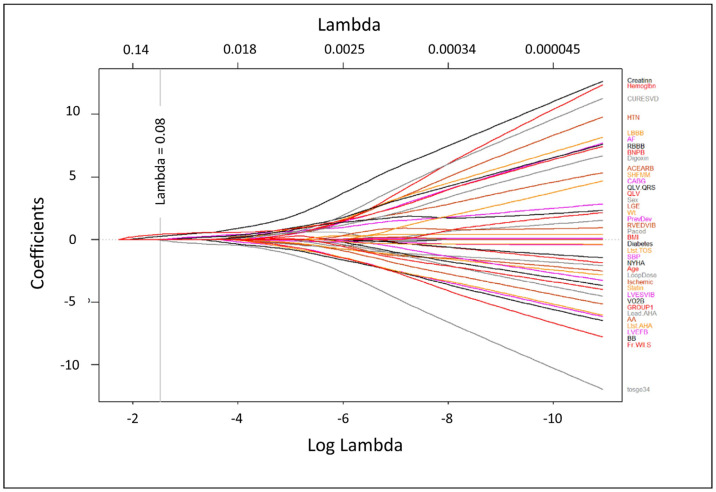
LASSO Coefficient Plot for Candidate Baseline Variables. The plot shows the candidate variable coefficients as a function of the log of the tuning parameter, λ. The variables with non-zero coefficients at a given value of the tuning parameter can be identified as those with curves that have not yet shrunk to zero. This plot identified B-type natriuretic peptide, creatinine, coronary artery bypass grafting, and CMR mechanical activation at the LVP site ≥34 ms as the best predictors of 4-year survival).

A plot of the LASSO regression coefficient values as a function of the tuning parameter (λ) is shown in Figure 5. Using a tuning parameter of 0.08, the following four parameters were identified for survival prediction: pre-CRT BNP, pre-CRT creatinine, history of CABG surgery, and a CMR activation time at the LV lead implant site ≥ 34 ms. Although Group 2 was associated with decreased 4-year survival, the other four covariates had more significant associations in the LASSO regression model. Of note, although patients in Group 2 were older, age was not a significant predictor of survival (*p* = 0.32).

### 3.5. Training and Test Sets with Standard Logistic Regression

The cohort was then split into a 70% training set and a 30% test set, and a standard logistic regression model with these four covariates was derived in just the training set. Predictions from this model in the test set were compared with actual events, and the predictions had an accuracy of 93.3% for predicting 4-year survival in the test set.

### 3.6. Receiver Operating Characteristic Analysis and Nomogram

**Figure 6:**
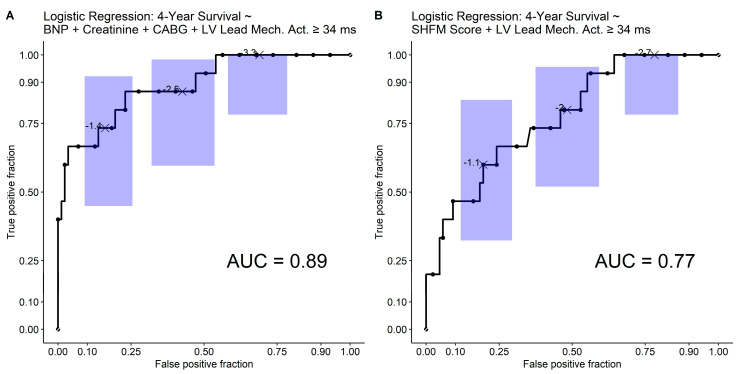
Receiver Operating Characteristic (ROC) Plots for 4-Year Survival. (**A**) The overall ROC plot for 4-year survival based on the covariates of baseline B-type natriuretic peptide, creatinine, prior coronary artery bypass grafting surgery, and mechanical activation ≥ 34 ms at the LV pacing site is shown with exact 95% confidence intervals at three points of interest according to the method of Pearson and Clopper. (**B**) For reference, the corresponding ROC plot with the SHFM score and mechanical activation > 34 ms at the LV pacing site as predictors is shown.

**Figure 7:**
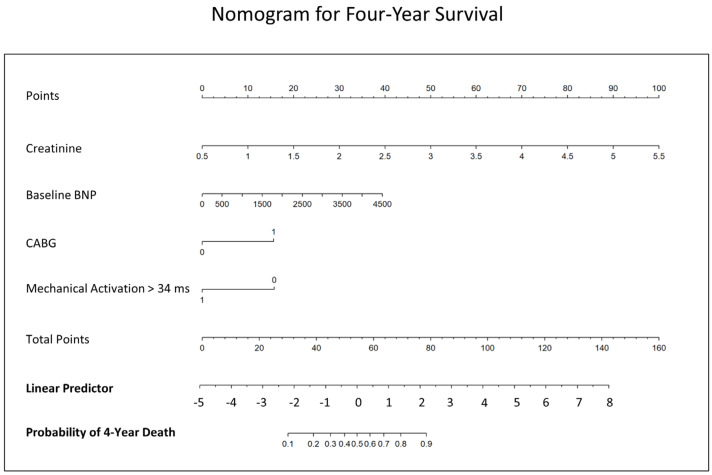
Nomogram for Prediction of 4-Year Survival Across Groups. The nomogram provides the probability of 4-year survival after CRT using covariates of pre-CRT B-type natriuretic peptide, pre-CRT creatinine, prior coronary artery bypass grafting, and CMR mechanical activation of at least 34 ms.

Receiver operating characteristic analysis from a logistic regression model with these four covariates in all 102 patients demonstrated a model AUC of 0.89 (*p* < 0.0001) (Figure 6A). The model is shown in Table S1. For clinical application, a nomogram to estimate 4-year survival based on this logistic regression model for 4-year survival is provided in Figure 7. For reference, a separate logistic regression model with two parameters, (1) the Seattle Heart Failure Model (SHFM) score substituted for the clinical covariates (BNP, creatinine, and prior CABG surgery) and (2) CMR activation time at the LV lead implant site ≥ 34 ms, had an AUC of 0.77 for 4-year survival (Figure 6B).

### 3.7. Kaplan–Meier Analysis and Cox Proportional Hazards Analysis

**Figure 8:**
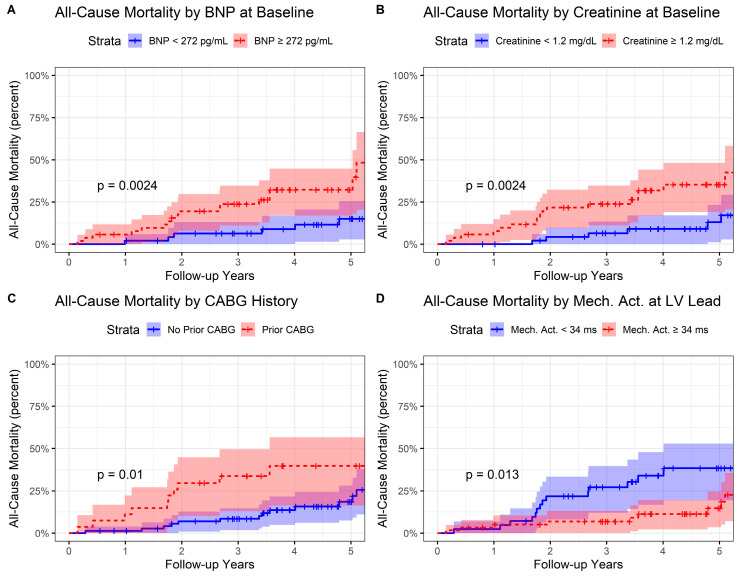
Reverse Kaplan–Meier Curves Stratified by Best Covariates for All-Cause Mortality. Reverse Kaplan–Meier curves are shown with stratification by: (**A**) baseline B-type natriuretic peptide stratified by the median value of 272 pg/mL; (**B**) baseline creatinine stratified by the median value of 1.2 mg/dL; (**C**) those with and without coronary artery bypass grafting; and (**D**) and those with CMR mechanical activation stratified by the optimal cutoff value of 34 ms.

In a Cox proportional hazards model with 26 death events, the pre-CRT creatinine, the pre-CRT BNP, CABG history, and mechanical activation ≥ 34 ms were all associated with survival in the multivariable model (Table S2). Creatinine had the greatest association with survival in this model with a hazard ratio HR of 5.78 per mg/dL (*p* < 0.0001). Baseline BNP (*p* = 0.012), CABG (*p* = 0.020), and CMR mechanical activation time ≥ 34 ms at the LV pacing site (*p* = 0.040) were also significant predictors in the model. Reverse Kaplan–Meier curves are shown in Figure 8 for baseline BNP stratified by the median (A), baseline creatinine stratified by the median (B), prior CABG (C), and mechanical activation ≥ 34 ms (D). For reference, the corresponding survival model with the SHFM score and CMR mechanical activation time ≥34 ms at the LV pacing site is shown in Table S3. The hazard ratio for the SHFM in this model was HR 2.81 per unit increase in SHFM score (*p* = 0.0059).

### 3.8. Mediation Effects

**Figure 9:**
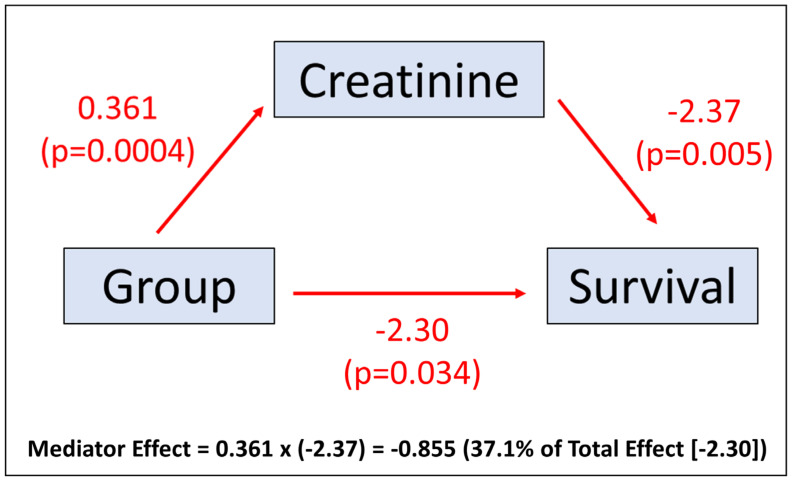
Mediation Plot. The mediation plot demonstrates the mediation effect of creatinine relative to the total effect from group assignment.

The effect of a CRT upgrade with RVP-HF relative to de novo CRT on the likelihood of survival was partially mediated by the pre-CRT creatinine. The regression coefficient for the total effect of the CRT upgrade with RVP-HF on survival time using parametric survival regression with a Gaussian distribution was −2.302 (*p* = 0.034). As Figure 9 illustrates, the causal mediation effect was (0.361) × (−2.37) = −0.855. The bootstrapped 95% confidence interval for the mediator effect was −1.89 to −0.19 (*p* = 0.008), and the bootstrapped confidence interval for the total effect was −3.85 to −0.25 (*p* = 0.034). There was a 37% mediation effect of creatinine for the RVP-HF group effect on survival time (−0.855/−2.302 = 0.371). Other candidate baseline characteristics and response measures did not have a significant average causal mediation effect.

## 4. Discussion

This study offers a novel presentation of differences in prognosis, CMR structural findings, comorbidities, neurohormonal activation, renal function, and electromechanical differences at the LV pacing site among patients undergoing CRT upgrades with and without baseline RV pacing dependence versus de novo CRT patients. An important clinical finding is that baseline renal function and levels of neurohormones are the predominant drivers of survival after both de novo CRT implants and CRT upgrades with a significant contribution from whether late CMR mechanical activation is achieved at the left ventricular (LV) pacing site. These findings also present a roadmap to identify prognosis in patients with CRT upgrades, and a nomogram is provided for this purpose. Moreover, the worse survival in upgrade RVP-HF patients in this cohort was mediated significantly by a comorbid condition, specifically renal impairment, in the upgrade RVP-HF cohort. Other comorbid conditions more common in CRT upgrade patients for RVP-HF in this cohort, such as atrial fibrillation, were found not to mediate the decreased survival as impaired kidney function did.

With respect to prognosis in CRT upgrades, prior studies have yielded partially conflicting results. While some studies found similar overall survival in de novo and upgrade CRT patients [24,25], others found that upgrade CRT patients had worse survival [22,23]. CRT upgrade patients were noted to have an increased prevalence of atrial fibrillation and more impaired kidney function, as we found in the present cohort. Furthermore, CRT upgrade patients with RVP-HF in our cohort had smaller baseline LV volumes compared with the CRT upgrade patients having other indications and de novo CRT patients. In many ways, upgrade patients without RVP-HF looked more like de novo patients than upgrade patients with RVP-HF, which highlights the importance of distinguishing upgrade patients with and without RV pacing dependence.

It is remarkable that the Budapest Upgrade CRT randomized trial showed a clinical benefit for upgrades to CRT devices in patients with RV pacing dependence [43]. The findings in our study are consistent with those from the Budapest Upgrade CRT trial because the comparison groups in our study for the patients with RV pacing dependence and CRT upgrades were patients with either de novo CRT implants or patients with CRT upgrades not dependent on RV pacing. In contrast, the control group for the Budapest Upgrade CRT trial consisted of patients with RV pacing dependence who did not receive CRT upgrades through randomization.

With respect to implant findings and prognosis, LV free wall sites having later mechanical and electrical activation timing represent the areas in the LV that are most dysfunctional and typically undergoing the most stretch in early systole, such that pacing these sites is likely to have the most beneficial effect on LV function. In this cohort, we found the electrical activation at LVP site, mechanical activation at the LVP site, and overall mechanical dyssynchrony based on the CURE-SVD mechanical dyssynchrony index were prognostically important across de novo CRT and upgrade groups. The finding that electrical and mechanical activation at the LVP site both contribute favorably to response and survival in this mixed cohort highlights the importance of achieving both late electrical and late mechanical activation to achieve optimal CRT response rates.

Another remarkable finding in this cohort is the anterior shift in late-activated sites observed for CRT upgrade patients with RVP-HF. This can be understood intuitively by the fact the RV lead position is typically in the area of seven o’clock on the LV short-axis views, and the anterior LV at the one o’clock position is the most distant from the RV pacing lead along the circumference of LV short axis, thus representing the most late-activated site. This anterior shift in late activation in RVP-HF has important implications for the CRT implant procedure in patients with CRT upgrades with RV pacing in this area. Although the LV pacing site chosen was at the discretion of the operator in this study, and this study was not a prospective interventional trial of CMR guidance for the selection of the LVP site, the prognostic importance of mechanical and electrical late activation at the LVP site in this cohort suggests that CRT upgrade patients with RVP-HF may have better results if operators target more anterolateral sites, and more anterior sites may even be good pacing targets in this scenario. In many patients, the anterior or anterolateral LV may be easier to access from the coronary sinus and may be less likely to have phrenic nerve capture; however, operators implanting CRT in patients with LBBB typically strive to achieve a more posterolateral LV lead position if there is not scar in this area. Furthermore, sometimes coronary venous pacing options are limited, and an anterior lead position may be the only one that works.

### Limitations

There were several limitations of this study. Although a cohort of 102 patients with curated CMR, clinical, laboratory, response, and survival data with a median follow-up over 4 years distributed over three groups is considered to be reasonably large and sufficient for the mechanistic study presented, a larger cohort would have facilitated additional approaches based on neural networks and other machine learning methods; however, it is reassuring that the main model had excellent accuracy in a test set when derived in a training set. Second, although patients were on maximally tolerated medical therapy appropriate at the time of enrollment, angiotensin-neprilysin inhibitors and sodium-glucose cotransporter-2 inhibitors have since become widely used medications for heart failure. While this is an inherent limitation for any long-term study because new medications may well be introduced during the five years of follow-up, the impact on the results of this study is likely very modest, as sodium-glucose cotransporter-2 inhibitors decrease heart failure and other cardiac events with minimal impact on survival [25], and heart failure risk models such as the SHFM have been shown to perform equally well in studies of angiotensin-neprilysin inhibitors [26] compared with other cohorts. Third, although conduction system pacing patients were not included in this analysis, the indication in heart failure with reduced ejection fraction is just now evolving, and the approach of incorporating CMR and neurohormonal/clinical data together with the statistical methodology presented in this paper can also be applied to future cohorts with conduction system pacing.

## 5. Conclusions

In conclusion, RVP-HF had a distinct CMR phenotype, which has important implications for the selection of LV pacing sites in CRT upgrades, and only chronic kidney disease mediated the decreased survival after CRT in RVP-HF.

## Figures and Tables

**Table 1:** Baseline Characteristics and CRT Response Outcomes by Group.

	All (N = 102)	De Novo CRT (N = 50)	CRT Upgrade with RVP (N = 29)	CRT Upgrade w/o RVP (N = 23)	*p* Value (All Groups)	*p* Value (Group 1 vs. 2)
**Demographics**						
Age, years	66.5 (58.3–72.9)	65.4 (60.4–70.8)	74.0 (64.0–79.0)	62.0 (56.0–70.0)	0.02	0.03
BMI, kg/m^2^	28.3 (24.2–33.2)	28.0 (24.0–31.7)	28.2 (23.3–33.1)	29.1 (25.4–35.5)	0.8	
Weight, kg	85.3 (75.3–103.0)	82.6 (73.6–99.8)	91.6 (73.9–103.0)	93.8 (80.2–103.9)	0.5	
Female	24 (23.5)	15 (30.0)	3 (10.3)	6 (26.1)	0.1	
NYHA Heart Failure Class					<0.0001	<0.0001
II	35 (34.3)	3 (6.0)	18 (62.1)	14 (60.9)		
III	67 (65.7)	47 (94.0)	11 (37.9)	9 (39.1)		
Race					0.5	
Black	16 (15.7)	6 (12.0)	5 (17.2)	5 (21.7)		
White/Other	86 (84.3)	44 (88.0)	24 (82.8)	18 (78.3)		
SHFM Score	0.38 (0.005–0.81)	0.55 (0.22–0.89)	0.19 (−0.01–0.79)	0.2 (−0.015–0.41)	0.1	
**Comorbid Conditions**						
Ischemic Cardiomyopathy	49 (48.0)	23 (46.0)	11 (37.9)	15 (65.2)	0.1	
Atrial Fibrillation	33 (32.4)	11 (22.0)	18 (62.1)	4 (17.4)	0.0004	0.0006
Chronic Kidney Disease	36 (35.3)	14 (28.0)	14 (48.3)	8 (34.8)	0.2	
Prior CABG	27 (26.5)	11 (22.0)	7 (24.1)	9 (39.1)	0.3	
**Medications**						
Beta-Blocker	96 (94.1)	49 (98.0)	25 (86.2)	22 (95.7)	0.07	
ACE Inhibitor/ARB	85 (83.3)	46 (92.0)	19 (65.5)	20 (87.0)	0.01	0.005
Loop Diuretic	79 (77.5)	40 (80.0)	18 (62.1)	21 (91.3)	0.04	0.1
Statin	67 (65.7)	28 (56.0)	19 (65.5)	20 (87.0)	0.04	0.5
**Laboratory Studies, Vital Signs & Exercise Testing**						
Systolic BP, mmHg	118.5 (102.0–130.0)	118.0 (108.0–129.8)	127.0 (110.0–138.0)	107.0 (96.5–120.0)	0.01	0.2
Sodium, mEq/L	138.0 (136.3–140.0)	138.0 (137.0–139.8)	139.0 (138.0–141.0)	137.0 (136.0–139.0)	0.1	
Creatinine, mg/dL	1.2 (0.96–1.4)	1.05 (0.9–1.3)	1.3 (1.2–1.6)	1.1 (0.95–1.4)	0.004	0.002
Hemoglobin, g/dL	13.3 (12.3–14.5)	13.6 (12.3–14.4)	13.1 (12.3–14.5)	13.1 (12.4–14.6)	0.9	
GFR, mL/min/1.72 m^2^	64.1 (51.5–82.6)	72.2 (59.1–87.0)	58.0 (43.0–74.0)	63.0 (54.0–77.0)	0.02	0.006
BNP, pg/mL	272.0 (137.3–752.8)	210.5 (118.3–699.0)	272.0 (124.0–879.0)	304.0 (262.0–642.0)	0.2	
Peak VO_2_, mL/kg/min	0.075 (−0.88–1.4)	14.3 (12.2–16.1)	14.4 (13.4–14.8)	14.4 (12.7–14.6)	0.9	
**CMR/Echocardiography** **Assessment Parameters**						
LVEF, %	23.7 (17.4–29.8)	23.2 (18.9–28.0)	29.0 (20.0–35.0)	19.0 (16.5–26.0)	0.02	0.06
LVEDVI, mL/m^2^	127.9 (104.8–165.0)	133.7 (112.2–170.2)	102.0 (77.4–115.0)	152.0 (126.2–183.5)	<0.0001	0.0002
LVESVI, mL/m^2^	97.2 (73.6–127.0)	101.2 (84.9–134.9)	71.3 (49.9–90.4)	117.0 (95.9–148.5)	<0.0001	0.0005
RVEF, %	36.1 (26.0–45.5)	36.1 (24.4–48.7)	37.5 (32.0–44.6)	35.1 (27.8–40.1)	0.7	
RVEDVI, mL/m^2^	65.8 (55.3–83.7)	66.0 (53.5–81.2)	65.3 (55.5–86.9)	65.8 (57.3–79.3)	0.9	
RVESVI, mL/m^2^	40.2 (32.7–55.2)	42.0 (31.8–57.7)	38.7 (31.6–52.8)	39.6 (38.1–50.3)	0.9	
LGE Presence	55 (53.9)	27 (54.0)	14 (48.3)	14 (60.9)	0.7	
CURE-SVD	0.57 (0.45–0.72)	0.60 (0.45–0.78)	0.55 (0.50–0.69)	0.55 (0.37–0.67)	0.4	
Scar at LV Free Wall	29 (28.4)	15 (30.0)	5 (17.2)	9 (39.1)	0.2	
**Electrical Parameters**						
Paced QRS	31 (30.4)	0 (0.0)	29 (100.0)	0 (0.0)	<0.0001	<0.0001
QRS, ms	161.0 (147.0–180.0)	160.0 (146.3–180.0)	179.0 (164.0–200.0)	152.0 (136.0–168.0)	0.0004	0.002
QLV, ms	130.0 (98.0–154.3)	112.5 (81.3–140.0)	160.0 (150.0–172.0)	108.0 (99.0–127.0)	<0.0001	<0.0001
QLV/QRS Ratio	0.78 (0.67–0.89)	0.74 (0.58–0.81)	0.89 (0.84–0.96)	0.76 (0.69–0.79)	<0.0001	<0.001
LBBB	67 (65.7)	48 (96.0)	0 (0)	16 (69.6)	<0.0001	<0.0001
RBBB	7 (6.9)	3 (6.0)	0 (0)	3 (13.0)	0.5	
TOS at LV Lead, ms	87.5 (69.0–114.8)	106.0 (72.3–118.0)	86.0 (66.0–107.0)	78.0 (68.0–96.5)	0.06	0.1
TOS at Latest Activated LV Segment, ms	119.5 (101.0–136.0)	124.0 (97.3–136.0)	112.0 (100.0–134.0)	119.0 (104.0–141.0)	0.7	
**Response Measures** **at 6 Months Post-CRT**						
Fractional Change in LVESVI	−0.2 (−0.32–−0.08)	−0.18 (−0.36–−0.023)	−0.21 (−0.36–−0.09)	−0.2 (−0.265–−0.135)	0.7	
BNP, pg/mL	172.5 (63.8–442.8)	132.5 (51.8–451.8)	172.5 (70.0–526.0)	195.0 (150.5–238.0)	0.6	
Change in Peak VO_2_, mL/kg/min	0.075 (−0.88–1.37)	0.40 (−1.5–2.3)	−0.26 (−0.9–0.4)	0.075 (−0.03–0.3)	0.3	
**Survival Status at 4 Years**					0.2	
Alive	83 (81.4)	44 (88.0)	21 (72.4)	18 (78.2)		
Dead	19 (18.6)	6 (12.0)	8 (27.6)	5 (21.8)		

Values are median (interquartile range) or n (%). ACE = angiotensin-converting enzyme; ARB = angiotensin receptor blocker; BMI = body mass index; BNP = B-type natriuretic peptide; BP = blood pressure; CABG = coronary artery bypass graft; CURE-SVD = circumferential uniformity ratio estimate with singular value decomposition; GFR = glomerular filtration rate; LBBB = left bundle branch block; LGE = late gadolinium enhancement; LVEDVI = left ventricular end-diastolic volume index; LVEF = left ventricular ejection fraction; LVESVI = left ventricular end-systolic volume index; NYHA = New York Heart Association; QLV = QRS-LV electrogram time; RBBB = right bundle branch block; RVEDVI = right ventricular end-diastolic volume index; RVEF = right ventricular ejection fraction; RVESVI = right ventricular end-systolic volume index; SHFM = Seattle Heart Failure Model; TOS = time to the onset of circumferential shortening.

**Table 2:** Locations of LV Lead Placement and Latest Mechanical Activation.

	De Novo CRT (N = 50)	CRT Upgrade with RVP (N = 29)	CRT Upgrade w/o RVP (N = 23)	*p* Value (All Groups)	*p* Value (Group 1 vs. 2)
**LV Lead Location**					
**Longitudinal**				0.4	
Basal	9 (18.0)	7 (24.1)	9 (39.1)		
Mid-ventricular	29 (58.0)	17 (58.6)	11 (47.8)		
Apical	12 (24.0)	5 (17.2)	3 (13.0)		
**Circumferential**				0.6	
Anterior	8 (16.0)	1 (3.4)	2 (8.7)		
Anteroseptal	0 (0.0)	0 (0.0)	0 (0.0)		
Inferoseptal	0 (0.0)	0 (0.0)	0 (0.0)		
Inferior	1 (2.0)	0 (0.0)	1 (4.3)		
Inferolateral	12 (24.0)	7 (24.1)	5 (21.7)		
Anterolateral	29 (58)	21 (72.4)	15 (65.2)		
**Location of Latest** **Mechanical Activation**					
**Longitudinal**				<0.0001	
Basal	14 (28.0)	25 (86.2)	16 (69.6)	<0.0001	<0.0001
Mid-ventricular	31 (62.0)	4 (13.8)	6 (26.1)	<0.0001	<0.0001
Apical	5 (10.0)	0 (0.0)	1 (4.3)	0.6	
**Circumferential**				0.003	
Anterior	5 (10.0)	14 (48.3)	9 (39.1)	0.0002	0.0002
Anteroseptal	0 (0.0)	0 (0.0)	0 (0.0)		
Inferoseptal	0 (0.0)	0 (0.0)	0 (0.0)		
Inferior	1 (2.0)	1 (3.4)	1 (4.3)	0.8	
Inferolateral	17 (34.0)	2 (6.9)	7 (30.4)	0.02	0.007
Anterolateral	27 (54)	12 (41.4)	6 (26.1)	0.08	

Values are n (%).

## Data Availability

The data for this study will be posted on GitHub.

## Supplementary Materials

The following supporting information can be downloaded at https://www.mdpi.com/article/10.3390/jcdd10100409/s1, Table S1: Supplemental Logistic Regression Model; Table S2: Supplemental Cox Proportional Hazards Regression Model #1; Table S3: Supplemental Cox Proportional Hazards Regression Model #2.
