# Supplementary material for: Cardiac Magnetic Resonance, Electromechanical Activation, Kidney Function, and Natriuretic Peptides in Cardiac Resynchronization Therapy Upgrades
Source: J Cardiovasc Dev Dis. 2023 Sep 22;10(10):409. doi: 10.3390/jcdd10100409 (PMC10607260; doi:10.3390/jcdd10100409)
Supplement: Supplementary file 1 [file jcdd-10-00409-s001.zip › jcdd-2562826-SI.pdf]

Table S1: Logistic Regression Model

```
Logistic Regression Model

                       Model Likelihood     Discrimination    Rank Discrim.
                             Ratio Test            Indexes          Indexes
Obs           102    LR chi2      34.33     R2       0.463    C       0.871
 0             83    d.f.             4     R2(4,102)0.257    Dxy     0.743
 1             19    Pr(> chi2) <0.0001    R2(4,46.4)0.480    gamma   0.743
max |deriv| 7e-07                           Brier    0.097    tau-a   0.227

           Coef    S.E.   Wald Z Pr(>|Z|)
Intercept  -4.4023 1.1999 -3.67  0.0002
BNPB        0.0008 0.0003  2.36  0.0180
Creatinine  1.8155 0.7447  2.44  0.0148
CABG        1.4198 0.6863  2.07  0.0386
CMR.MAGE34 -1.4343 0.6943 -2.07  0.0388
```

Table S2: Cox Proportional Hazards Regression Model #1

```
Cox Proportional Hazards Model #1

  n= 102, number of events= 26

                 coef  exp(coef)   se(coef)      z Pr(>|z|)
Creatinine  1.7545023  5.7805701  0.3907157  4.490 7.11e-06 ***
BNPB        0.0004698  1.0004699  0.0001878  2.502   0.0124 *
CABG        1.0960313  2.9922671  0.4697240  2.333   0.0196 *
CMR.MAGE34 -0.9403157  0.3905045  0.4583849 -2.051   0.0402 *
---
Signif. codes:  0 ‘***’ 0.001 ‘**’ 0.01 ‘*’ 0.05 ‘.’ 0.1 ‘ ’ 1

           exp(coef) exp(-coef) lower .95 upper .95
Creatinine    5.7806     0.1730     2.688    12.432
BNPB          1.0005     0.9995     1.000     1.001
CABG          2.9923     0.3342     1.192     7.513
CMR.MAGE34    0.3905     2.5608     0.159     0.959

Concordance= 0.817  (se = 0.044 )
Likelihood ratio test= 42.64  on 4 df,   p=1e-08
Wald test            = 35.9  on 4 df,   p=3e-07
Score (logrank) test = 67.14  on 4 df,   p=9e-14
```

Table S3: Cox Proportional Hazards Model #2

```
Cox Proportional Hazards Model #2

  n= 102, number of events= 26

             coef    exp(coef) se(coef)    z  Pr(>|z|)
SHFMM       1.0319    2.8064   0.3744  2.756  0.00585 **
CMR.MAGE34 -0.8043    0.4474   0.4075 -1.974  0.04843 *
---
Signif. codes:  0 '***' 0.001 '**' 0.01 '*' 0.05 '.' 0.1 ' ' 1

         exp(coef) exp(-coef) lower .95 upper .95
SHFMM       2.8064     0.3563    1.3473    5.8458
CMR.MAGE34  0.4474     2.2351    0.2013    0.9945

Concordance= 0.709  (se = 0.052 )
Likelihood ratio test= 13.45  on 2 df,   p=0.001
Wald test            = 12.91  on 2 df,   p=0.002
Score (logrank) test = 13.65  on 2 df,   p=0.001
```
